# Magnetic Resonance Imaging Allows the Evaluation of Tissue Damage and Regeneration in a Mouse Model of Critical Limb Ischemia

**DOI:** 10.1371/journal.pone.0142111

**Published:** 2015-11-10

**Authors:** Germana Zaccagnini, Anna Palmisano, Tamara Canu, Biagina Maimone, Francesco M. Lo Russo, Federico Ambrogi, Carlo Gaetano, Francesco De Cobelli, Alessandro Del Maschio, Antonio Esposito, Fabio Martelli

**Affiliations:** 1 Molecular Cardiology Laboratory, IRCCS-Policlinico San Donato, San Donato Milanese, Milan, Italy; 2 Preclinical Imaging Facility, Experimental Imaging Center, San Raffaele Scientific Institute, Milan, Italy; 3 Vita-Salute San Raffaele University, Milan, Italy; 4 Department of Clinical Sciences and Community Health, University of Milan, Milan, Italy; 5 Division of Cardiovascular Epigenetics, Department of Cardiology, Internal Medicine Clinic III, Goethe University, Frankfurt am Main, Germany; University of Rome La Sapienza, ITALY

## Abstract

Magnetic resonance imaging (MRI) provides non-invasive, repetitive measures in the same individual, allowing the study of a physio-pathological event over time. In this study, we tested the performance of 7 Tesla multi-parametric MRI to monitor the dynamic changes of mouse skeletal muscle injury and regeneration upon acute ischemia induced by femoral artery dissection. T2-mapping (T2 relaxation time), diffusion-tensor imaging (Fractional Anisotropy) and perfusion by Dynamic Contrast-Enhanced MRI (K-trans) were measured and imaging results were correlated with histological morphometric analysis in both Gastrocnemius and Tibialis anterior muscles. We found that tissue damage positively correlated with T2-relaxation time, while myofiber regeneration and capillary density positively correlated with Fractional Anisotropy. Interestingly, K-trans positively correlated with capillary density. Accordingly, repeated MRI measurements between day 1 and day 28 after surgery in ischemic muscles showed that: 1) T2-relaxation time rapidly increased upon ischemia and then gradually declined, returning almost to basal level in the last phases of the regeneration process; 2) Fractional Anisotropy dropped upon ischemic damage induction and then recovered along with muscle regeneration and neoangiogenesis; 3) K-trans reached a minimum upon ischemia, then progressively recovered. Overall, Gastrocnemius and Tibialis anterior muscles displayed similar patterns of MRI parameters dynamic, with more marked responses and less variability in Tibialis anterior. We conclude that MRI provides quantitative information about both tissue damage after ischemia and the subsequent vascular and muscle regeneration, accounting for the differences between subjects and, within the same individual, between different muscles.

## Introduction

Critical limb ischemia (CLI) is the most severe form of peripheral artery disease, is mostly caused by atherosclerosis, and is characterized by intractable pain, ulcers and gangrene which require a prompt treatment [1]. A large inter-individual variability in the severity of the injury following the ischemic event has been observed between different patients. Indeed, up to a third of CLI patients are not amenable to conventional intervention, such as angioplasty and surgical bypass, and also those who benefit from successful revascularization, suffer from high rates of recurrent symptoms or revision surgery and many still require progressive amputations [1, 2]. Given the morbidity, mortality, and costs associated with CLI, optimal treatment to increase limb preservation, prevent death, and improve functional capacity in these patients is still a unmet need [3]. In this context, preclinical studies, designed to understand the mechanisms underpinning cellular and tissue response to ischemia or to test the efficacy of prospective therapeutic agents, could provide new therapeutic options. Specifically, different mouse models of acute hindlimb ischemia have been developed to mimic at least certain aspects of human CLI: among different options, femoral artery excision results in a profound reduction in blood flow to the ischemic limb, even at rest, closely mimicking the clinical condition of CLI patients [2, 4, 5]. Due to blood flow deprivation or reduction, mouse skeletal muscle architecture undergoes dramatic changes. A first phase of tissue damage, characterized by apoptosis, necrosis, inflammation, edema and decreased capillary density, is followed, with some overlapping, by a process of tissue repair including angiogenesis and skeletal muscle regeneration [6–8]. Of note, a rather high variability in the outcome of the ischemic injury has been observed between different mouse strains and between mice of the same strain, due to genetic and microvascular morphology differences [9, 10]. This is particularly true in CD1 mice, an outbred mouse strain widely used as mouse model of ischemic injury [11–17]. On the one side, this inter-individual variability models the heterogeneity observed in humans; on the other side, it can complicate the analysis and increase number of mice necessary to obtain statistically solid data.

Therefore, the identification of a non-invasive imaging method, able to identify and quantitatively monitor over time the biological phenomena occurring after ischemia, is needed. Magnetic Resonance Imaging (MRI) provides an excellent qualitative evaluation of soft tissue. In addition, recent preclinical studies highlighted the ability of advanced quantitative MRI techniques to describe non-invasively the dynamic modifications of skeletal muscle in different models of damage and regeneration [18–30]. Indeed, the modification of T2 relaxation time (T2-rt) may reflect the degree of muscle damage and inflammatory infiltration, while Diffusion-tensor imaging (DTI) may provide information about muscle micro-architectural modification and myofibre regeneration [18, 23, 31]. However, the dynamic events taking place during ischemic damage are still incompletely characterized. In particular, the identification of imaging biomarkers of microvascular modifications that occurs after ischemia may be useful to non-invasively quantify the severity of ischemia and also to monitor the response to experimental therapies. In this regard, a recent study conducted in a small population of diabetic patients affected by peripheral microangiopathy suggests that dynamic contrast enhancement (DCE-MRI) assessment of K-trans may provide a non-invasive quantitative evaluations of perfusion in Tibialis anterior muscle [32].

In this study, we tested the performance of 7 Tesla multi-parametric MRI in investigating *in vivo* the dynamic changes of CD1 mouse skeletal muscles after femoral artery excision, by correlating the imaging results with histological analysis. We observed that MRI provides a comprehensive and panoramic identification of the events taking places during the damage-healing process in ischemic limb and shows the heterogeneous involvement of different muscles.

## Materials and Methods

### Animal model of hindlimb ischemia

All experimental procedures complied with the Guidelines of the Italian National Institutes of Health and with the *Guide for the Care and Use of Laboratory Animals* (Institute of Laboratory Animal Resources, National Academy of Sciences, Bethesda, Md), were approved by the Institutional Animal Care and Use Committee of San Raffaele Scientific Institute, Milan, Italy and were authorized by “Ministero della Salute" (IACUC n°551).

**Fig 1:**
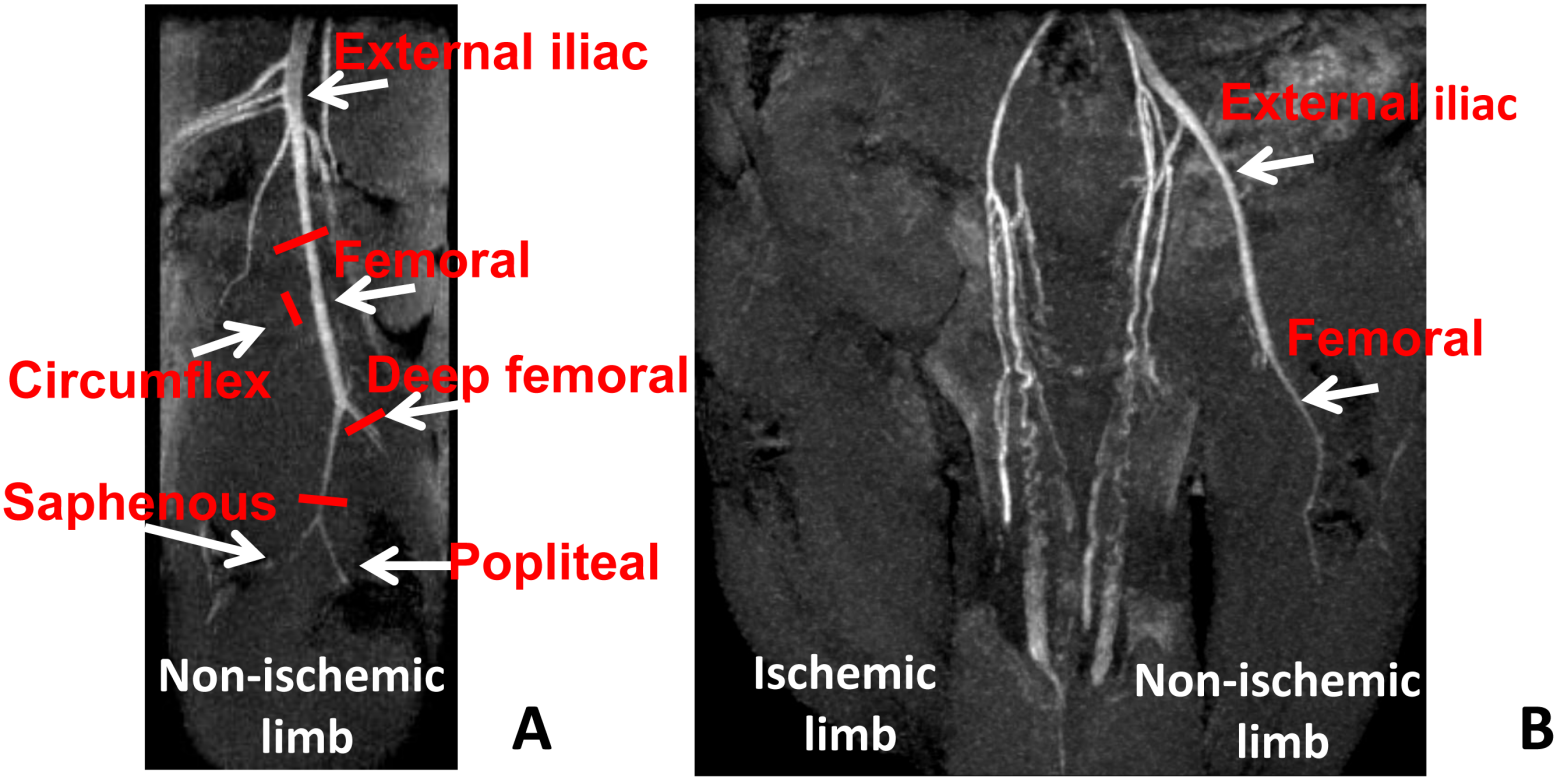
Magnetic resonance angiography (MRA) confirms the efficacy of the femoral artery dissection procedure. Coronal and sagittal MIP reconstructions of representative mouse limbs MRA, 1 day after femoral artery dissection. **A)** Non-ischemic right limb (sagittal plane). The femoral artery, its major branches circumflex and deep femoral and the bifurcation in saphenous and popliteal, are shown. Red bars indicate the sites in which arteries were electro-cauterized and cut during the surgery in the opposite hindlimb. **B)** Ischemic (left) and non-ischemic (right) limbs 1 day after surgery (coronal plane). MRA shows both external iliac and femoral arteries in the right non-ischemic limb; conversely they were undetectable in the left limb, demonstrating that surgery was carried out successfully.

Two months old CD1 male mice were used. Before all surgical and perfusion procedures, mice were anesthetized with an intraperitoneal injection of 10 mg/kg Xilazine (Intervet Farmaceutici) and 100 mg/kg Ketamine (Ketavet 100; Intervet Farmaceutici). Acute hindlimb ischemia was induced by removing the femoral artery as previously described [33]. Specifically, after hair removal, the mouse was placed in supine position under a stereomicroscope (Stemi 2000-C, Zeiss) and the left limb was extended and secured. The skin was wiped with betadine and incised from the knee towards the medial thigh for approximately 1 cm. The subcutaneous fat pad was put distally for a better exposure. The left femoral artery was dissected free from the nerve and the vein immediately below the inguinal ligament and was occluded and cut by a bipolar electro-cauterizer. Then, the whole artery and its major branches, circumflex and deep femoral, were dissected free from the vein and the nerve, occluded and cut. The distal electro-cauterization was performed just before the bifurcation in saphenous and popliteal artery (Fig 1A). The skin incision was closed by a silk suture 5/0 Ethicon.

### Magnetic resonance imaging

MRI studies were performed using a 7T preclinical magnetic resonance scanner (Bruker, BioSpec 70/30 USR, Paravision 5.0, equipped with 450/675 mT/m gradients (slew-rate: 3400–4500T/m/s; rise-time: 140ms). A volumetric body coil was used as receiver. Mice were imaged under general anesthesia obtained by 1.5–2% isoflurane (ForaneH, Abbott) vaporized in 100% oxygen (flow: 1l/min), in prone position, with both inferior limbs aligned on axial plane. During the examination, breathing rate and body temperature were monitored (SA Instruments, Inc.) and maintained stable around 40 breaths per minute and 37°C, respectively. A field-map based shimming (MAPSHIM software package, ParaVision-5.0, Bruker) was performed to optimize B0 field homogeneity before imaging. MRI protocol included T2-mapping, DTI and DCE-MRI. T2-maps were obtained using a Multi-Slice-Multi-Echo (MSME) sequence with fat suppression (repetition-time = 3000 ms; 12 echo-times = 8.30–99.60 ms; field-of-view = 32 x 15 mm; matrix = 158 x 100; spatial resolution = 0.203 x 0.150 mm/pixel; NSA = 2) acquired on axial plane (6 slices; thickness = 1 mm; gap = 0 mm). DTI was performed using a SpinEcho-EPI sequence (DTI-Epi) with 30 diffusion gradient directions (repetition-time = 2800 ms; echo-time = 22.56 ms; b-values for direction = 870 s/mm4; diffusion gradient duration = 5 ms; diffusion gradient separation = 10 ms; NSA = 2), acquired on axial plane (field-of-view = 33x15 mm; spatial resolution = 0.258x0.117 mm; 6 slices; slice thickness = 1 mm; gap = 0 mm). DCE-MRI was performed acquiring a 2D Flash dynamic sequence with 120 dynamics (repetition-time = 3000 ms; echo-times = 3 ms; flip angle 70°, field-of-view = 220 x 150 mm; spatial resolution = 0.250 x 0.117 mm; temporal resolution 6 sec; NA 1) during automatic injection of 5 μL of Gd-DTPA (MAGNEVIST, Bayer) at an injection rate of 0.5 μL/sec.

Additionally, a hindlimb Magnetic Resonance Angiography (MRA) was performed at day 1 for the assessment of femoral artery dissection. MRA was carried out with a 3D FLASH sequence (repetition-time = 20 ms; echo-time = 3.14 ms; flip_angle 15° NSA = 1) acquired on the coronal plane (field-of-view = 35x35x9 mm; matrix size 256x256x192 spatial resolution = 0.137 x 0.137 x 0.047 mm/pixel)

#### Image analysis

MRI post-processing was performed with Paravision-5.0 software (Bruker). Average FA, and T2-rt values were obtained from the regions of interest (ROIs) positioned on Tibialis anterior and Gastrocnemius muscles in 3 subsequent slices. ROIs were also positioned on Tibialis anterior and Gastrocnemius of healthy non injured limb used as internal control. DCE-MRI analysis were performed using DCE@urLAB software [34] and volume transfer coefficient (K-trans) pixel-by-pixel maps were obtained using region-of-reference model. MRA studies were reconstructed in the coronal and sagittal planes using maximum intensity projection (MIP) method.

### Histology and morphometric analysis

Mice were perfused via the left ventricle with phosphate-buffered saline (PBS), followed by 10% buffered formalin, at 100 mmHg for 10 min. Next, Gastrocnemius and Tibialis anterior muscles were harvested, fixed, and paraffin embedded. Sections of 3μm thickness were prepare and stained. Morphometric analysis was carried out on Hematoxylin/Eosine stained sections [8]. Briefly, sections were deparaffinized in Xilene, rehydrate in descending alcohol solutions, stained in Hematoxilin solution (50% Mayer’s Hematoxilin, 50% Carazzi’s Hematoxilin, Bio-Optica) for 4 min and rinsed with tap water for 10 minutes to allow the staining to develop. Next, sections were washed in deionized water and stained with Eosin-Phloxin B alcoholic-acid solution (Eosin Y cat. n. E-4382 Sigma Aldrich; Phloxin B cat. n. P40-30 Sigma Aldrich) for 1 minute. After washing in deionized water, sections were de-hydrate in ascending alcohol solution, cleared 3 time in Xilene and mounted by synthetic based mounting medium (Bio-mount 05-BM250 Bio-Optica). For morphometric analysis, damaged tissue including infiltrating cells and necrotic muscle fibers was identified by morphology, differential Eosin staining, and presence of infiltrating cells into and near the degenerating fibers. Regenerating myofibers were identified by the presence of a central nucleus and a small diameter (20–35μm) during the early regeneration phase and by multiple central nuclei and larger diameter during the late regeneration process. Areas of damaged, regenerating or adipose tissue were quantified in 25–35 random fields of the whole section of Gastrocnemius muscles and 10–12 random fields/section of Tibialis anterior muscles at 200x magnification [7]. Capillary density was measured counting the number of capillary profiles in 20–40 random fields/section, at 1000x magnification [8]. α—smooth muscle actin (α—SMA) labeling was used to identify arterioles. After deparaffinization, and rehydration, muscle sections were stained with α—SMA antibody (α—SMA clone 1A4; Sigma) diluted 1:30 in Antibody diluent with background reducing components (S3022, Dako) for one hour at 37°C. Afterwards, sections were rinsed with PBS, incubated with anti-mouse IgG Fab specific FITC conjugate secondary antibody (F5262 Sigma, final dilution 1:30) for 1 hour at 37°C, rinsed again and coverslipped with VectaMount AQ (H5501 Vector Laboratories). Arterioles, with at least one layer of stained smooth muscle cells, were visualized at 40x magnification. Images were acquired on the whole section and arterioles were classified on the basis of the minimum internal diameter in three different ranges: 4–10.99 μm, 11–20.99 μm and 21–40.99 μm. The arteriolar length density (LD) was determined by the following formula: LD (mm/mm3) = Σ (a/ b)/M, where a and b are the maximum and minimum internal arteriolar diameters, respectively, and M is the solid tissue area [35].

A Zeiss Imager M1 fluorescence microscope with image analyzer Axiovision Release 4.8.2 software was used to acquire images and to measure areas. All histological and morphometric analyses were carried out by two blinded readers with comparable results.

### Statistical analysis

Continuous variables are expressed as mean ± standard error (SE) unless indicated differently. All statistical tests were performed 2-sided and a p <0.05 was considered as statistically significant. For group-wise comparisons, statistical analysis was performed using 2-tail Student’s t-test. MRI and morphometric measurements correlations were analyzed by Pearson or Spearman tests, as appropriate, using GraphPad Prism v.4.03 (GraphPad Software Inc.) and by the Principal Component Analysis.

The longitudinal profiles of MRI measurements T2-rt, FA and K-trans through time were investigated using mixed effects linear models. In particular the comparison between ischemic and non-ischemic Gastrocnemius muscles and between ischemic and non-ischemic Tibialis anterior muscles were considered for T2-rt, FA and K-trans. In addition, the comparison between ischemic Gastrocnemius and ischemic Tibialis anterior muscles was performed for T2-rt, FA and K-trans normalizing the values of ischemic muscles using the values obtained from the non-ischemic contralateral limbs. Thus, a time specific trend for each muscle (ischemic/non-ischemic, Gastrocnemious/Tibialis anterior) was inserted into the model through an interaction with time. In particular, a random intercept (mouse as grouping factor) and random slope (for time effect) model was used. The possible non-linear effect of time was investigated using natural splines. Likelihood ratio tests were used for model selection. The longitudinal profiles predicted by the mixed effects regression model (with 95% confidence intervals) were reported in figures together with observed mean values at each time point. Moreover, the differences at time 0 and the temporal trend for each muscle was estimated according to mixed regression models [36]. The free software R was used for mixed effects models and principal component analysis [37].

## Results

### MRI validation by histological analysis

**Fig 2:**
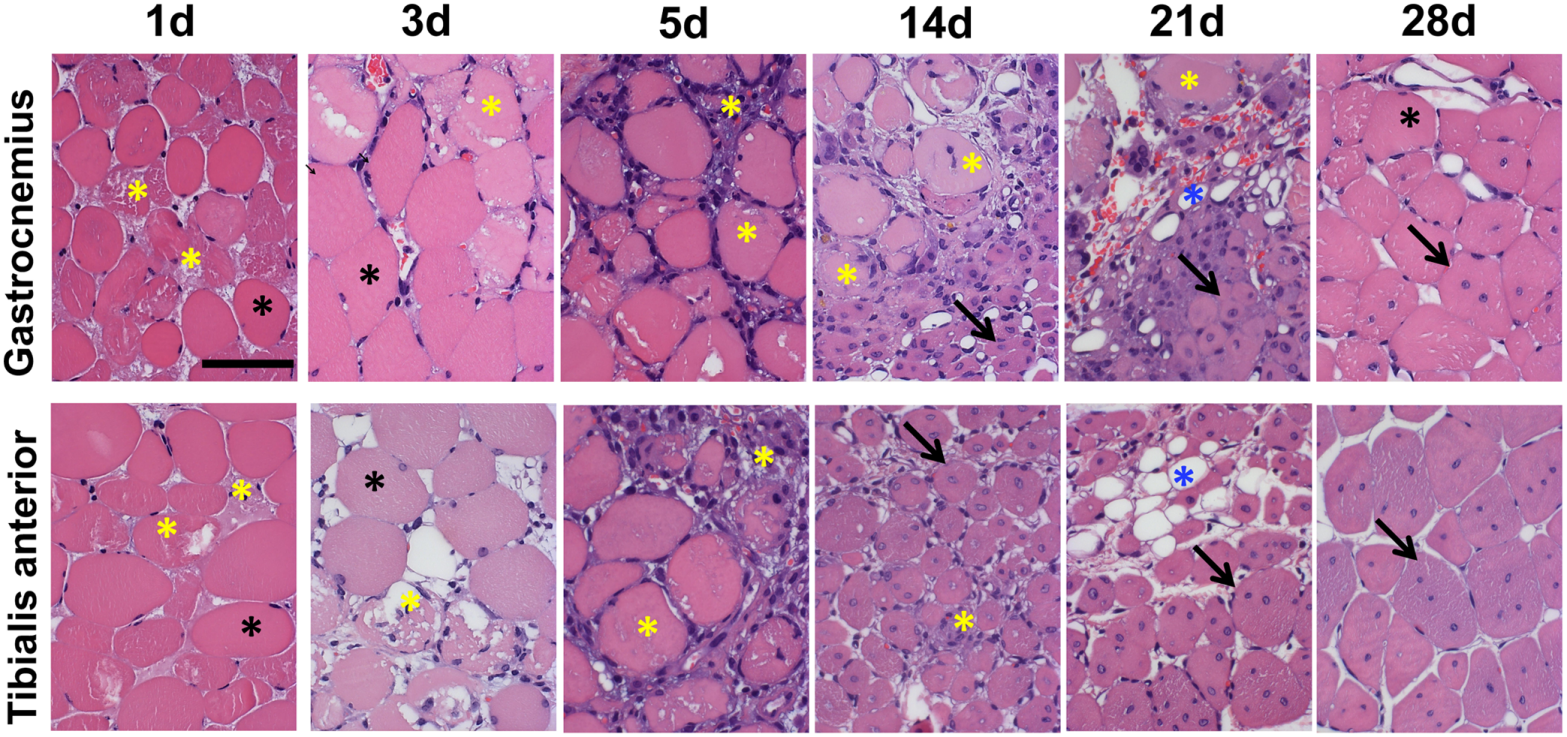
Morphometric analysis. Representative Hematoxilin/Eosin stained sections of Gastrocnemius (top) and Tibialis anterior muscles (bottom) following ischemia. Calibration bar = 50 μm. Black asterisks indicate healthy myofibers with peripheral nuclei. Yellow asterisks indicate damaged tissue, including necrotic myofibers and inflammatory infiltrating cells. Necrotic myofibers were identified by differential eosin staining and presence of infiltrating cells inside the fiber. Blue asterisks indicate adipose cells. Black arrows indicate regenerating myofibers characterized by the central nucleus/i.

To validate multi-parametric MRI as a tool to investigate the structural and functional changes caused by acute ischemia, MRI data were compared to morphometric analysis of histological sections. To this aim, the left femoral artery of 2 months old CD1 male mice was dissected free and removed (Fig 1A). Efficacy of the procedure was assessed in each mouse by MRA study performed at 1 day after surgery (Fig 1B). Ischemic mice were divided in 7 different groups and each of them underwent multi-parametric MRI at 1, 3, 5, 7, 14, 21 (N = 3) and 28 (N = 8) days after surgery (Table 1). T2-rt, FA and K-trans of Gastrocnemius and Tibialis anterior muscles of both posterior limbs were evaluated at each time point. Immediately after imaging, mice were sacrificed, and the muscles of ischemic and controlateral limbs were harvested for histological analysis. Thereafter, morphometric analysis was performed in Hematoxilin/Eosin stained sections (Fig 2). As expected, upon femoral artery dissection, areas of tissue damage were present from day 1 in both muscles, reaching a peak at day 5 and decreasing afterwards (S1A and S1B Fig). Muscle fiber regeneration started at day 7 in both muscles, reaching a peak respectively at 21 days in the Gastrocnemius and at day 28 in Tibialis anterior. The presence of small deposits of adipose tissue was also observed during the healing process, mainly in Tibialis anterior muscles (S1A and S1B Fig). Capillary density decreased immediately after surgery and reached a minimum at day 5 in both muscles, in agreement with the maximal tissue damage observed. Afterwards, it started to recover, reaching a peak at day 21 (S2 Fig). Arteriolar length density (ALD) showed no significant differences over time for both muscles (S3 Fig), possible due to the presence of a residual blood perfusion sufficient to preserve arteriole anatomical integrity [6].

**Table 1:** Experimental design for MRI/histological analysis correlation study.

T0	Day 1	Day 3	Day 5	Day 7	Day 14	Day 21	Day 28
Ischemia	MRI Histology	MRI Histology	MRI Histology	MRI Histology	MRI Histology	MRI Histology	MRI Histology

CD1 male mice underwent femoral artery dissection at T0. Next, they were divided in 7 groups, n = 3 mice for each time point between days 1 and 21, and n = 8 at day 28. MRI data were collected immediately before the sacrifice, followed by Gastrocnemius and Tibialis anterior muscles harvesting for histological analysis.

**Fig 3:**
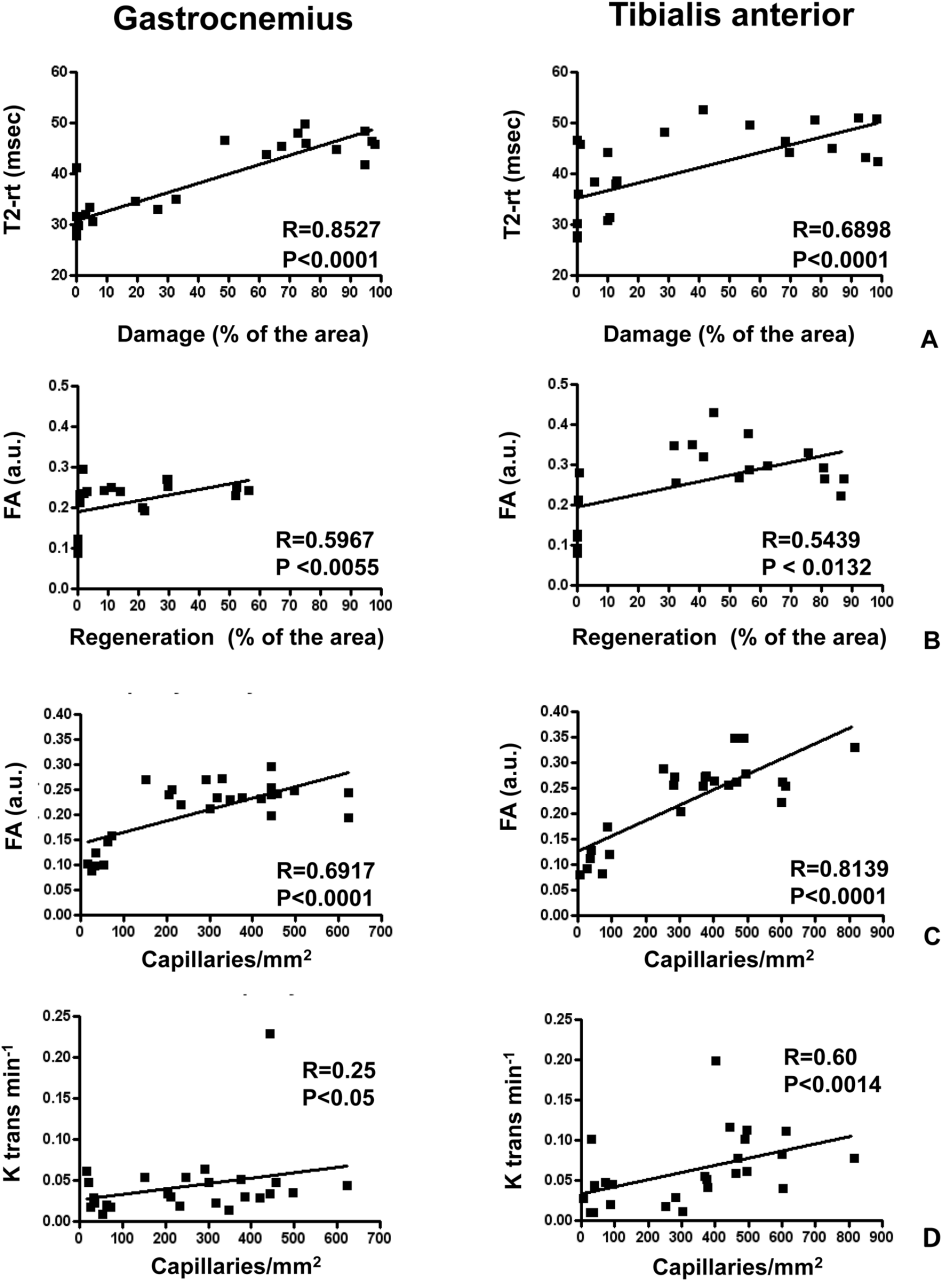
Correlations between histological changes and MRI parameters. The graphs represent the correlations between histological results and MRI measurements in ischemic Gastrocnemius (left panels) and ischemic Tibialis anterior (right panels) muscles. Each dot represents one mouse. **A**) Correlation analysis between percentage area of damaged tissue and T2-rt values. **B)** Correlation analysis between the percentage area of regenerating tissue and FA. **D** and **E)** Correlation analysis between capillary density and both FA, and K-trans, respectively.

The correlation analysis of the MRI parameters with morphometry performed at different time points, showed that tissue damage positively correlated with T2-rt (Fig 3A) and myofiber regeneration positively correlated with FA (Fig 3B), both in Gastrocnemious and in Tibialis anterior muscle. Interestingly, a correlation between capillary density and both FA and K-trans was also found (Fig 3C and 3D). These correlations were confirmed using an independent analysis method (Principal Component Analysis, not shown). ALD did not correlate with any MRI parameter in both Gastrocnemius and Tibialis anterior muscles, as expected given the minimal changes observed.

Taken together these results demonstrated that, also in a mouse model of femoral artery dissection, multi-parametric MRI can quantitatively monitor the biological events occurring in skeletal muscle after acute ischemic damage.

### Characterization of CD1 response to ischemia by MRI

**Fig 4:**
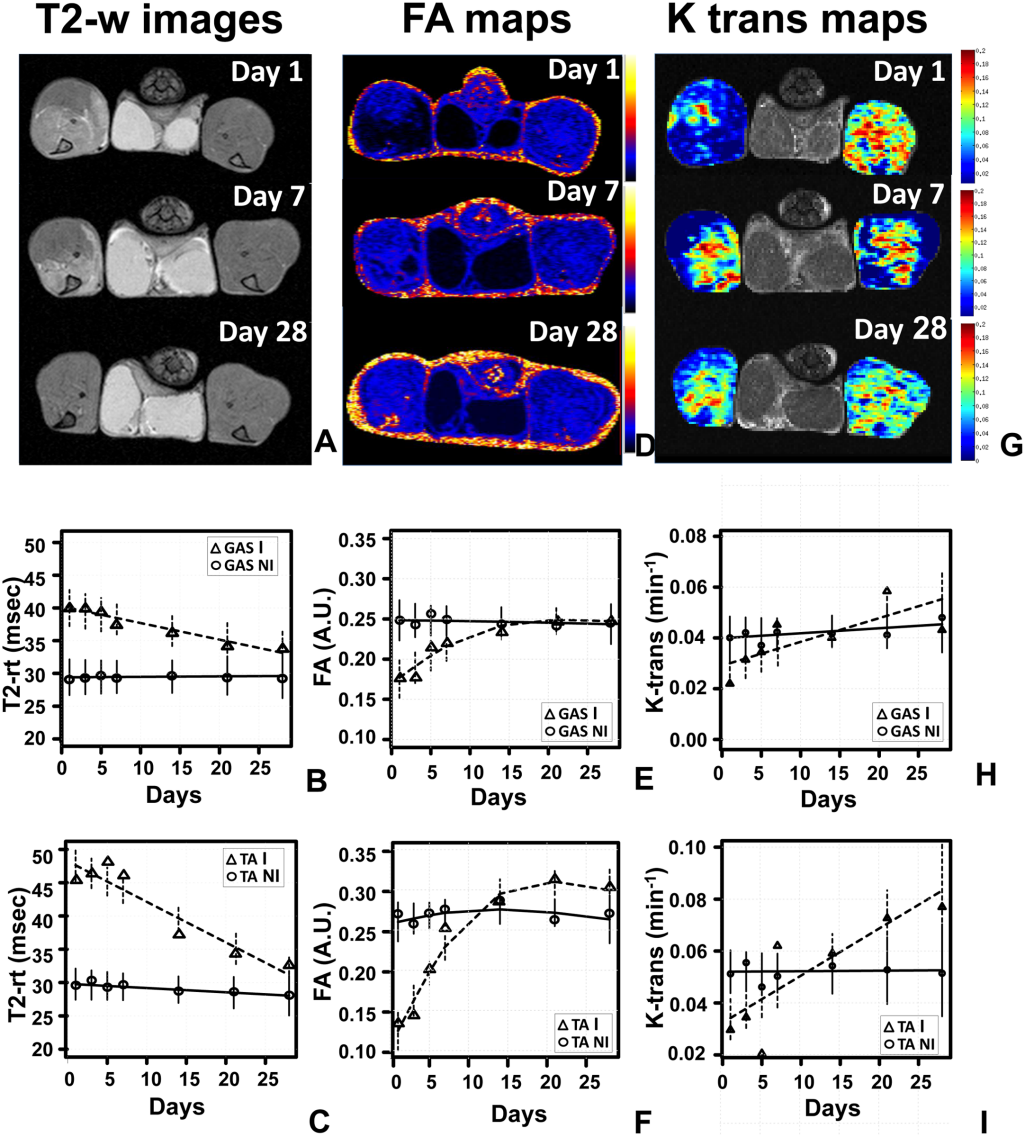
Muscle T2-rt, FA and K-trans evaluation after acute hindlimb ischemia. Representative T2-weighted images (**A**), FA maps (**D**) and K-trans maps (**G**) obtained at 1, 7 and 28 days after femoral artery dissection showing the heterogeneous involvement of the ischemic muscles and the modification of each parameter during the healing process. Time trend analysis of T2-rt, FA and K-trans are shown in panels B, C, E, F, H and I. Mean observed (circle: non-ischemic muscles; triangle: ischemic muscles) and model estimates (continuous line: non-ischemic muscles; broken line: ischemic muscles) are reported for the different time points. Vertical bars represents 95% confidence intervals according to the mixed regression model. Gas: Gastrocnemius muscle; TA: Tibialis anterior muscle. **B** and **C)** The graphs show the time trend analysis of T2-rt in Gastrocnemius and Tibialis anterior respectively, providing evidence for a different temporal trend between ischemic and non-ischemic muscles (interaction with time: Gastrocnemius p<0.01; Tibialis Anterior p<0.001). For non-ischemic muscles the temporal trend appeared constant. T2-rt levels were significantly increased at day 1 (both Gastrocnemius and Tibialis anterior p<0.001). **E)** and **F)** The graphs show the time trend analysis of FA in Gastrocnemius and Tibialis anterior respectively, providing evidence for a different non-linear temporal trend between ischemic and non-ischemic muscles (interaction with time p<0.001 for both Gastrocnemius and Tibialis anterior). For non-ischemic muscles the temporal trend were constant. FA levels were significantly decreased at day 1 (both Gastrocnemius and Tibialis anterior p<0.001) then started to converge. **H** and **I)** The graphs show the time trend analysis of K-trans in Gastrocnemius and Tibialis anterior respectively, providing evidence for a difference between ischemic and non-ischemic Tibialis anterior muscles (interaction with time, p<0.001). No evidence for a different temporal trend between ischemic and non-ischemic muscles was found for Gastrocnemius (p = 0.065). For Non Ischemic, muscles the temporal trend appeared constant. K-trans levels at day 1 were significantly decreased in Tibialis anterior (p = 0.002) then increased until day 28.

MRI allows repeated measurements in the same individual, providing data on the evolution of the ischemia response in different muscles of the limb. This can be particularly useful when mouse strains with high variability, such as CD1, are analyzed. Taking advantage of this MRI feature, the modifications of skeletal muscle architecture in response to the ischemic injury were studied in a time course by repeated multi-parametric MRI at 1, 3, 5, 7, 14, 21 (N = 11) and 28 (N = 8) days after surgery (Table 2). T2-rt, FA and perfusion by DCE-MRI (K-trans) were measured and muscles of the healthy contralateral limbs were used as internal controls (Fig 4A, 4D and 4G; S1 Table). The analysis over time of T2-rt, FA and K-trans of Gastrocnemius and Tibialis anterior muscles highlighted a clear-cut different temporal trend between ischemic and non-ischemic muscles (Fig 4B–4I). T2-rt levels were significantly increased at day 1 for both ischemic muscles respect to non-ischemic, then started to decrease, returning to almost basal level at day 28. The T2-rt interaction with time was significant for both muscles, while for non-ischemic muscles, the temporal trend appeared constant (Fig 4A, 4B and 4C). FA levels of ischemic muscles compared to non-ischemic were significantly decreased at day 1, then slowly returned to basal levels following a non- linear trend, while for non-ischemic muscles, the temporal trend appeared constant (Fig 4D, 4E and 4F). The K-trans interaction with time was significant for ischemic Tibialis anterior muscle respect to non-ischemic, but not for Gastrocnemius muscles, showing non linearity through time. However, K-trans levels displayed greater variability compared to other MRI measures (Fig 4G, 4H and 4I).

**Table 2:** MRI time course design.

T0	Day 1	Day 3	Day 5	Day 7	Day 14	Day 21	Day 28
Ischemia	MRI	MRI	MRI	MRI	MRI	MRI	MRI

CD1 ischemic mice underwent repeated MRI at the indicated times after surgery and were sacrificed at the end of the time course (n = 3 mice at day 21 and n = 8 mice at day 28).

Taken together these results showed a higher susceptibility and a stronger response of Tibialis anterior muscle compared to Gastrocnemius to the ischemic insult.

**Fig 5:**
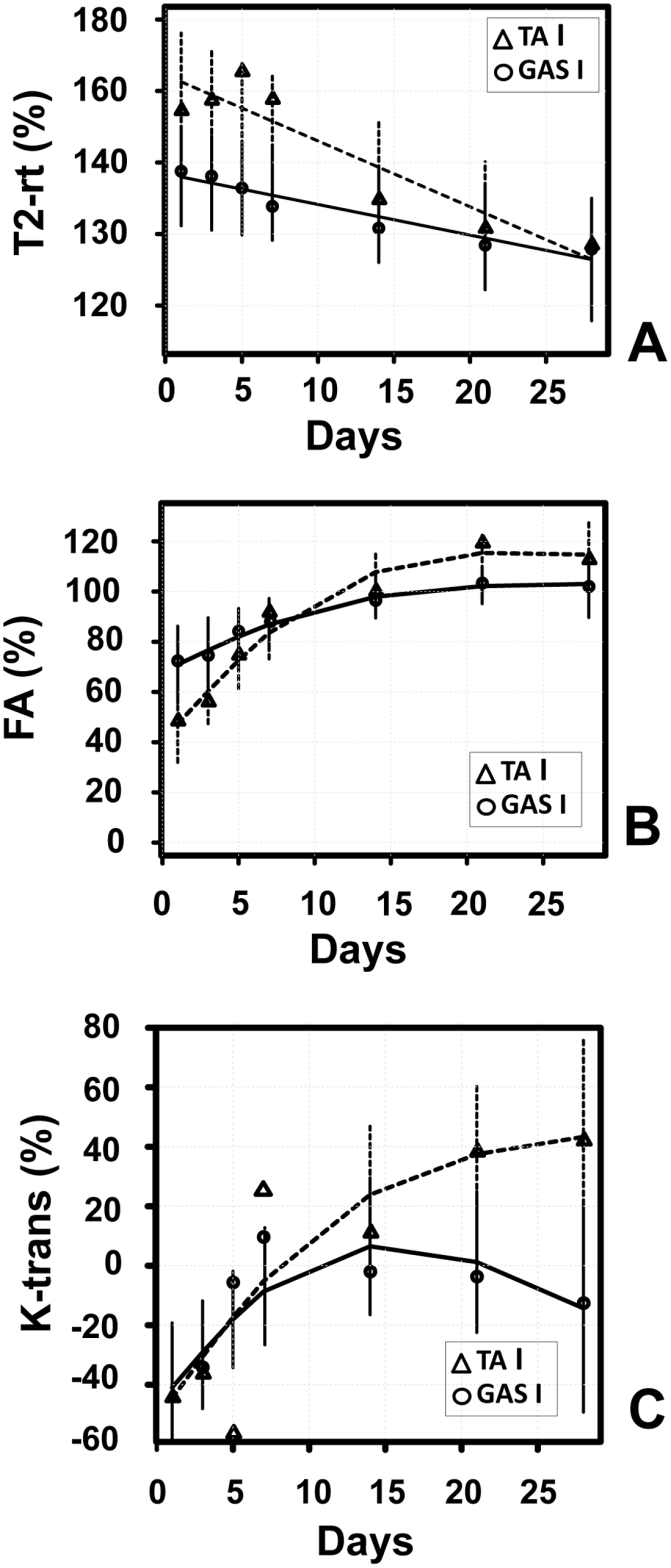
Longitudinal profiles of MRI measurements through time. Time trend analysis of T2-rt, FA and K-trans levels for Gastrocnemius and Tibialis anterior muscles. All values were normalized to values measured in non-ischemic limbs. Mean observed (circle: Gastrocnemius; triangle: Tibialis anterior) and model estimates (continuous line: Gastrocnemius; broken line: Tibialis anterior) are reported. Vertical bars represents 95% confidence intervals according to the mixed regression model. Evidence of a different time trend between the two muscles was found for all MRI measurements (interaction with time T2-rt: p = 0.03; FA: p<0.001; K-trans: p = 0.03). Evidence of a non-linear trend was found for both FA and K-trans. The recovery rate of Tibialis anterior appeared always greater than that of Gastrocnemius. **A)** T2-rt (ischemic/contralateral*100); **B)** FA (ischemic/contralateral*100); **C)** K-trans [(Ischemic-controlateral)/controlateral*100].

To highlight these differences, the temporal trend of Tibialis anterior and Gastrocnemius muscles were normalized and directly compared (Fig 5). The longitudinal analysis of ischemic/contralateral T2-rt ratios provided evidence for a different temporal trend between Gastrocnemius and Tibialis anterior. T2-rt levels at day 1 were significantly different and then started to decrease, but at a rate about double for Tibialis anterior compared to Gastrocnemius muscle (Fig 5A). Likewise, a different temporal trend between Gastrocnemius and Tibialis anterior was found also for FA. The difference of the drop at day 1 of FA between Gastrocnemius and TA was statistically significant. When FA started to recover, the recovery rate of Tibialis anterior was always greater than that of Gastrocnemius and both rates attenuated after day 7 (Fig 5B). The longitudinal analysis of K-trans levels also provided evidence for a different temporal trend between Gastrocnemius and Tibialis anterior (Fig 5C).

These results show that the muscles of the tibialis region of the limb are differentially affected by femoral artery excision in CD1 mouse strain.

### Low-response phenotype assessment

**Fig 6:**
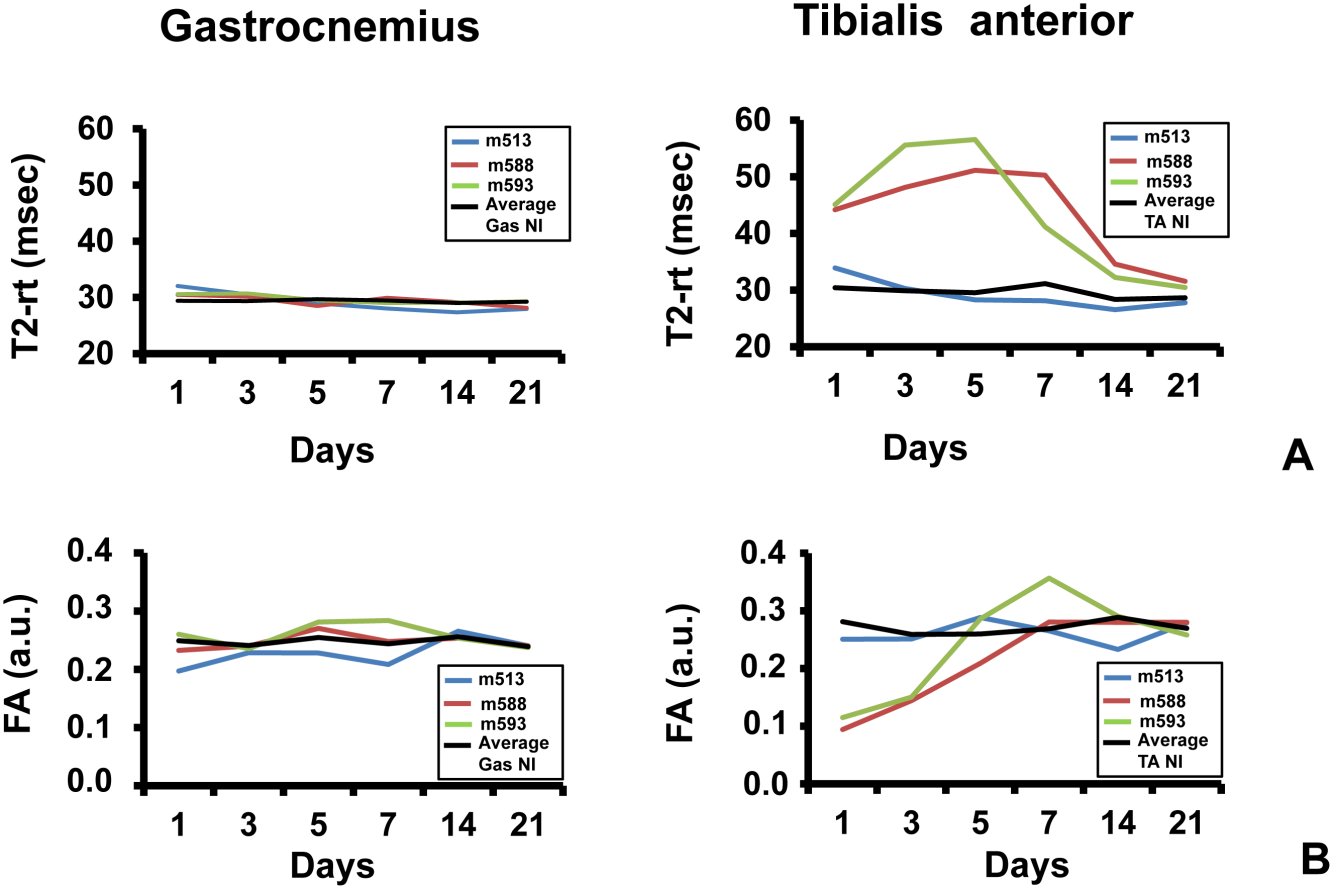
Low-response, ischemic resistant phenotype assessment. The graphs show the dynamic trend of MRI parameters of 3 mice out of 11 which displayed an atypical, “low-response” phenotype. Each mouse was identified by an ID: m513 (blue), m588 (red) and m593 (green). Average values measured in the controlateral non-ischemic limbs were used as reference (black). **A)** T2-rt (msec) of Gastrocnemius (right graph) and Tibialis anterior (left graph). **B)** FA (arbitrary units) of Gastrocnemius (right graph) and Tibialis anterior (left graph).

**Fig 7:**
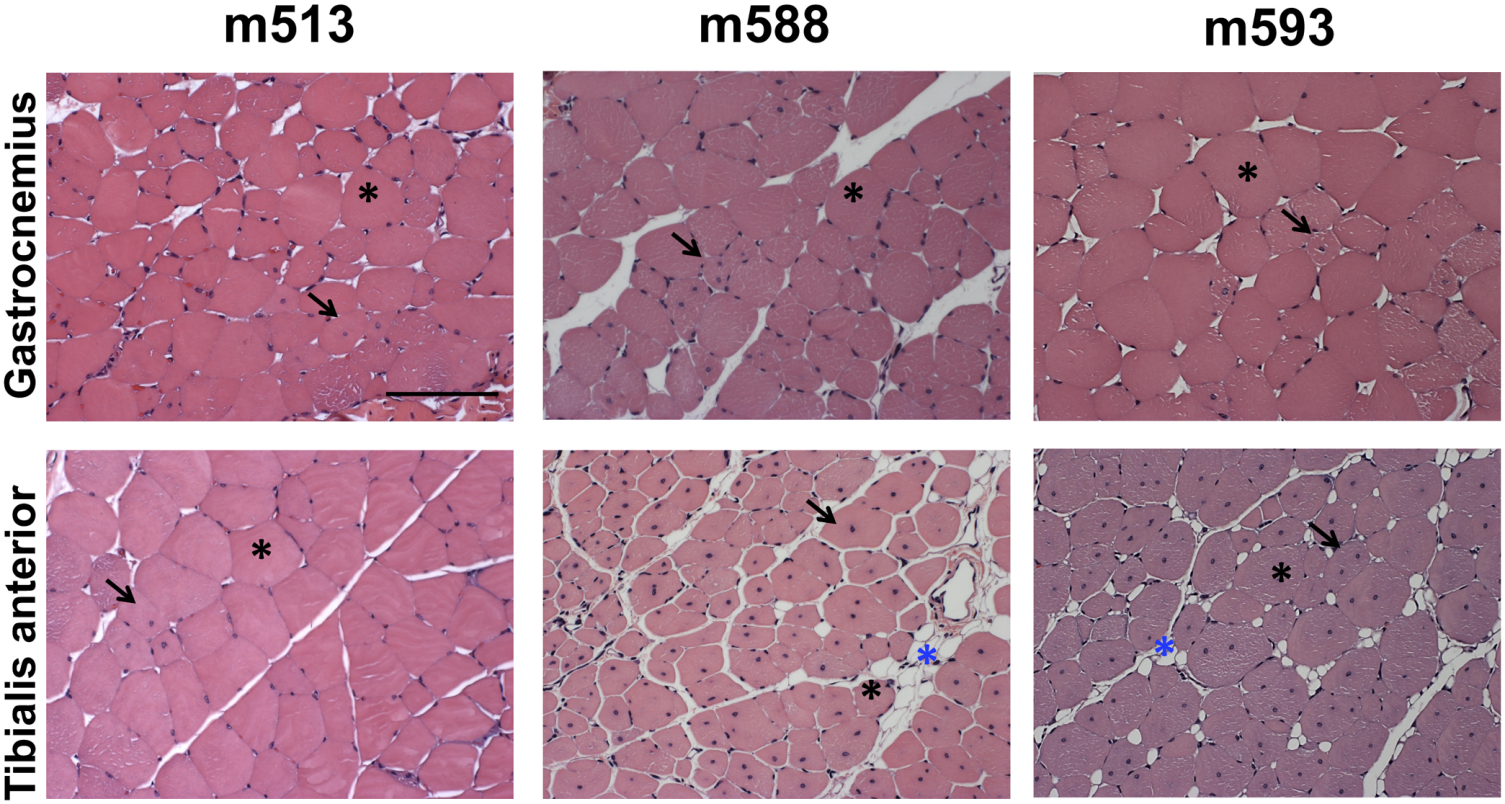
Muscle histological analysis of low-responding, ischemia resistant mice. Representative Hematoxilin/Eosin stained sections of both Gastrocnemius (top) and Tibialis anterior (bottom) muscles of mice m513, m588 and m593 at day 21 (m513) and 28 (m588 and m593) after ischemia. Calibration bar = 100 μm. Black asterisks indicate healthy myofibers with peripheral nuclei. Blue asterisks indicates adipose cells. Black arrows indicate regenerating myofibers characterized by the central nuclei.

To further investigate the different response of Gastrocnemius and Tibialis anterior muscles, we compared the dynamic trend of MRI parameters in each mouse. Interestingly we found that 3 mice out of 11 displayed an atypical, “low-response”, ischemic resistant phenotype, despite the surgery was carried out successfully, as assessed by MRA (S4 Fig). Mice m588 and m593 showed very similar T2-rt and FA parameters in the ischemic and in the controlateral Gastrocnemius muscles throughout the time course after femoral artery dissection (Fig 6A and 6B). On the contrary, these parameters measured in the Tibialis anterior muscle of the same limb, were altered as expected. When Hematoxilin/Eosin stained sections were analyzed at the end of the time course (Fig 7), important differences in the morphometric parameters were observed (Table 3). In particular, in keeping with the flat T2-rt curve, Gastrocnemius muscles did not show necrosis or tissue damage. Likewise, in agreement with the minimal changes observed in FA over time, only few regenerating myofibers were present in a globally healthy tissue. On the contrary, Tibialis anterior muscles displayed the expected post-ischemic pattern, with high percentage of regenerating myofibers and low adipose tissue substitution. Intriguingly, both Gastrocnemius and Tibialis anterior muscles displayed decreased capillary density (Table 3).

**Table 3:** Morphometric analysis of low-responding mice.

	Capillaries/mm^2^		% Damage		% Adipose tissue		% Regeneration	
Mouse ID	Gas	TA	Gas	TA	Gas	TA	Gas	TA
**m513**	444	496	0.0	0.0	0.0	0.0	2.9	0.7
**m588**	458	376	0.0	0.0	0.0	6.5	8.6	87.4
**m593**	375	402	0.0	0.0	0.0	5.0	2.1	80.9

Morphometric analysis of both Gastrocnemius (Gas) and Tibialis anterior (TA) muscles of mice m513, m588 and m593. The table shows the capillary density and the percentage area of damaged, adipose and regenerating tissue.

Finally, mouse m513 displayed a low-response pattern of MRI parameters (Fig 6) and histological features (Fig 7 and Table 3) in both Gastrocnemius and Tibialis anterior muscles, indicating that an attenuated response to ischemia can be also observed in Tibialis anterior and is not typical of the Gastrocnemius muscle.

## Discussion

In this study, we tested the performance of a multiparametric MRI protocol performed at 7T in investigating the dynamic changes of CD1 mouse skeletal muscles after femoral artery excision, by correlating the imaging results with data obtained from histological analysis. We demonstrated that, in this mouse model of hindlimb ischemia, multi-parametric MRI is a useful, sensitive and quantitative tool, allowing the non-invasive investigation of both tissue damage and regeneration. Our findings are in agreements with previous observations in different mouse models of damage/regeneration, both ischemic [11, 19] and non- ischemic [18, 23, 24]. Specifically, in both Gastrocnemius and Tibialis anterior muscles T2-rt increased immediately after surgery and then slowly decreased, returning almost to basal values at day 28 (Fig 4A, 4B and 4C and S1 Table). An increase in T2-rt lengthening is commonly considered a MRI sign of muscle damage reflecting severe changes in muscle architecture due to necrosis, edema and inflammation [38–41]. Accordingly, we observed a positive correlation between T2-rt and ischemic damage. Interestingly, a similar T2-rt dynamic trend was described by Gianella et al. [11] in Gastrocnemius muscle of CD1 mice after femoral artery dissection. An increased T2-rt was also described by Heemskerk et al. [19] in a mouse model of femoral artery ligation in C57BL/6 mice. The authors showed a T2-rt curve with a peak at day 3 followed by a rapid decline to basal value at day 21. This different trend and timing respect to our study is probably due to the milder damage induced by femoral artery ligation compared to excision. Additionally, the regeneration potential between CD1 and C57BL/6 inbred mice might differ. In a non-ischemic model of skeletal muscle damage-regeneration induced by cardiotoxin injection in Tibialis anterior muscles, Esposito and collaborators [23] described a rapid increase in T2-rt with a peak at day 3, followed by a progressive decrease to basal values within 30 days. A possible explanation for this difference in the T2-rt dynamic trend is that tissue damage induced by cardiotoxin is more localized, with persistence of a healthy vascular network and surrounding myofibers. On the contrary, femoral artery dissection leads to an extensive tissue damage, involving all the component of the skeletal muscle, with a consequent slower healing process. Another important difference between these two studies is that we used CD1 mice while Esposito et al. used of C57BL/6 mice. However, also in this model a positive correlation between muscle damage and T2-rt was observed, further strengthening our interpretation of T2-rt changes.

In intact skeletal muscle, intracellular water moves preferentially along the myofibers, due to the highly organized fibrillar structure. This anisotropic diffusion is quantified by the Epi-DTI sequence, measuring the FA [42]. The breakage of the myofibers during skeletal muscle damage, leads to a reduction of FA, due to the loss of a preferential direction of water molecules diffusion. On the contrary, during regeneration, FA progressively increases, possibly exceeding the basal value [23]. In agreement with these reports, we also observed a positive correlation between FA and skeletal muscle regeneration in both Tibialis anterior and Gastrocnemius muscles. In our study, FA of Gastrocnemius dropped at day 1 and then started to recover, overlapping the basal level from day 14. Tibialis Anterior muscles showed a similar trend until day 7; next, the recovery was faster than in Gastrocnemius and FA significantly exceeded the basal value at day 21 and 28 (Fig 4D, 4E and 4F and S1 Table). This dynamic trend of FA obtained in Tibialis anterior muscle after ischemia is similar to that observed by Esposito et al. after cardiotoxin injection; however the recovery was slower after ischemia, probably due to the higher severity of tissue damage and, consequently, to the different rate of regeneration.

The overall agreement of this study with previous literature on the interpretation of MRI parameters further demonstrates the importance of MRI as a tool for ischemic diseases research. However, important differences in the kinetics of MRI parameters changes have been observed, highlighting the importance of a careful condition set-up for each experimental model.

To the best of our knowledge, this is the first preclinical study in which the dynamic modification of K-trans was analyzed in a setting of acute ischemic damage. K-trans is the most commonly used quantitative parameter obtained from DCE-MRI data. Its main application is in oncological imaging, to assess the biological response to therapies having microcirculation as main target. K-trans changes may have different meaning according to the tissue studied and the pathophysiological conditions: in particular, K-trans may express either tissue permeability or blood flow, according to the factor limiting the Gadolinium diffusion within the tissue interstitial space [43]. In the adopted mouse model of acute hindlimb ischemia, the main factor conditioning the observed K-trans drop 1 day after injury is most likely the reduction in blood flow; accordingly, the following K-trans progressive increase is likely linked to the progressive restoration of the blood flow due to the regeneration of vessels and capillary network (Fig 4G, 4H and 4I and S1 Table). This interpretation is also supported by the observed correlation between K-trans and capillary density. Moreover, the K-trans values measured at later timepoints, especially in the Tibialis anterior, were above the threshold of the contralateral control muscle. This result may indicate an increased permeability, probably due to a number of factors, including immaturity of newly formed vessels and increase vasopermeability linked to residual inflammatory phenomena. Interestingly, the trend of modification of K-trans values observed after acute ischemic damage resulted to be very similar to the trend of modification of FA, suggesting a parallelism between the restoration of microcirculation and of the anisotropic muscle microarchitecture.

We also characterized the differences in the response to ischemia between different muscles. We observed that Tibialis anterior displayed more profound alterations and a lower number of low-responders to ischemia, indicating Tibialis anterior as the preferential site of analysis for ischemic studies in many circumstances. The reasons for the observed variability are unknown, however, the arteriolar network morphology pre-existing the ischemic insult may be an important determinant. Indeed, Gabhann and collaborators studied the microvascular phenotype of Spinotrapezius muscle in C57BL/6, Balb C inbred mice and CD1 outbred mice [10]. They found that, inbred mouse strains show two different angio-architectures: a heavily ramified arcade structure of the arteriolar network, which correlates with low levels of tissue damage upon ischemia; alternatively, a dendritic and unconnected arteriolar trees in which each input arteriole feeds an independent vessel system, which correlates with high levels of damage. Interestingly, CD1 outbred mice displayed an intermediate phenotype, with a higher inter-individual variability in the angio-architecture morphology then inbred strains.

MRI demonstrated to be a very useful complement of histological analysis, providing accurate and quantitative data, with the significant advantage of allowing non-invasive, repetitive measures in the same individual. This is particular important when later stages of the regenerative process are analyzed. Indeed, in the presence of a minimally altered histological pattern, MRI allows to distinguish between those mice that, after damage, underwent an almost complete regeneration process, and those low-responders that exhibited very little damage in the early phases. This aspect is particularly important to facilitate data interpretation and to minimize the numerosity of the experimental groups, in agreement with the current principles of the animal experimentation. Indeed, the use of MRI for the characterization of mouse hindlimb ischemia models is in full agreement with the “Reduction and Refinement” principles indicated in the Directive 2010/63/EU on the protection of animals used for scientific purposes, and adopted in all scientifically advanced countries.

In conclusion MRI allows to non-invasively and quantitatively investigate not only microvascular changes and overt skeletal muscles responses to femoral artery excision, but also subtle differences in the susceptibility of each muscle to the ischemic insult.

## Supporting Information

S1 Fig Morphometric analysis of tissue damage and repair. Gastrocnemius and Tibialis anterior muscles were harvested at the indicated times after Femoral artery dissection. Morphometric analysis was carried out on Hematoxilin/Eosin stained sections. The bar graphs show the percentage areas of necrotic, adipose or regenerating tissue. **A**) Gastrocnemius muscles; **B**) Tibialis anterior muscles.
(TIF)

S2 Fig Capillary density. The bar graph represents the quantification of capillaries/mm^2^ of both Gastrocnemius (Gas) and Tibialis anterior (TA) muscles.
(TIF)

S3 Fig Arteriolar Length density. The bar ghraphs represent the ALD of Gastrocnemius (A) and Tibialis anterior (B) muscles. Arterioles were identified by α—smooth muscle actin (α—SMA) staining and were classified on the basis of the minimum external diameter in three different ranges: 4–10.99 μm, 11–20.99 μm and 21–40.99 μm. No statistically significant differences were observed (n = 3).
(TIF)

S4 Fig MRA of low-responding, ischemia resistant, mice. The pictures show coronal 3D FLASH images of both ischemic and non-ischemic limb of low-responding mice performed at 1 day after surgery. External Iliac (white arrows) and femoral artery (red arrows) are readily detectable in the right non-ischemic limb and completely undecteable in the left ischemic limb, demonstrating that surgery was carried out successfully in each ischemia resistant mice. **A)** Mouse m513; **B)** Mouse m588; **C)** Mouse m593.
(TIF)

S1 Table MRI measurements. MRI parameters of Tibialis anterior (TA) and Gastrocnemius (Gas) muscles, both ischemic (I) and non-ischemic (NI) measured after femoral artery dissection. Results are reported as average values of T2-rt (msec), FA (arbitrary units) and K-trans (min^-1^). The statistical significance (P) was calculated by Student’s T test comparing ischemic and non-ischemic controlateral muscles.
(PDF)

## Abbreviations

ALD: Arteriolar Length Density
CLI: Critical limb ischemia
DCE-MRI: Dynamic contrast enhanced Magnetic Resonance Imaging
DTI: Diffusion-tensor imaging
DTI-Epi: SpinEcho-EPI sequence
FA: Fractional anisotropy
MRI: Magnetic Resonance Imaging
MSME: Multi-Slice-Multi-Echo
T2-rt: T2 relaxation time
