# Supplementary figures and images for: Magnetic Resonance Imaging Allows the Evaluation of Tissue Damage and Regeneration in a Mouse Model of Critical Limb Ischemia
Source: PLoS One. 2015 Nov 10;10(11):e0142111. doi: 10.1371/journal.pone.0142111 (PMC4640853; doi:10.1371/journal.pone.0142111)

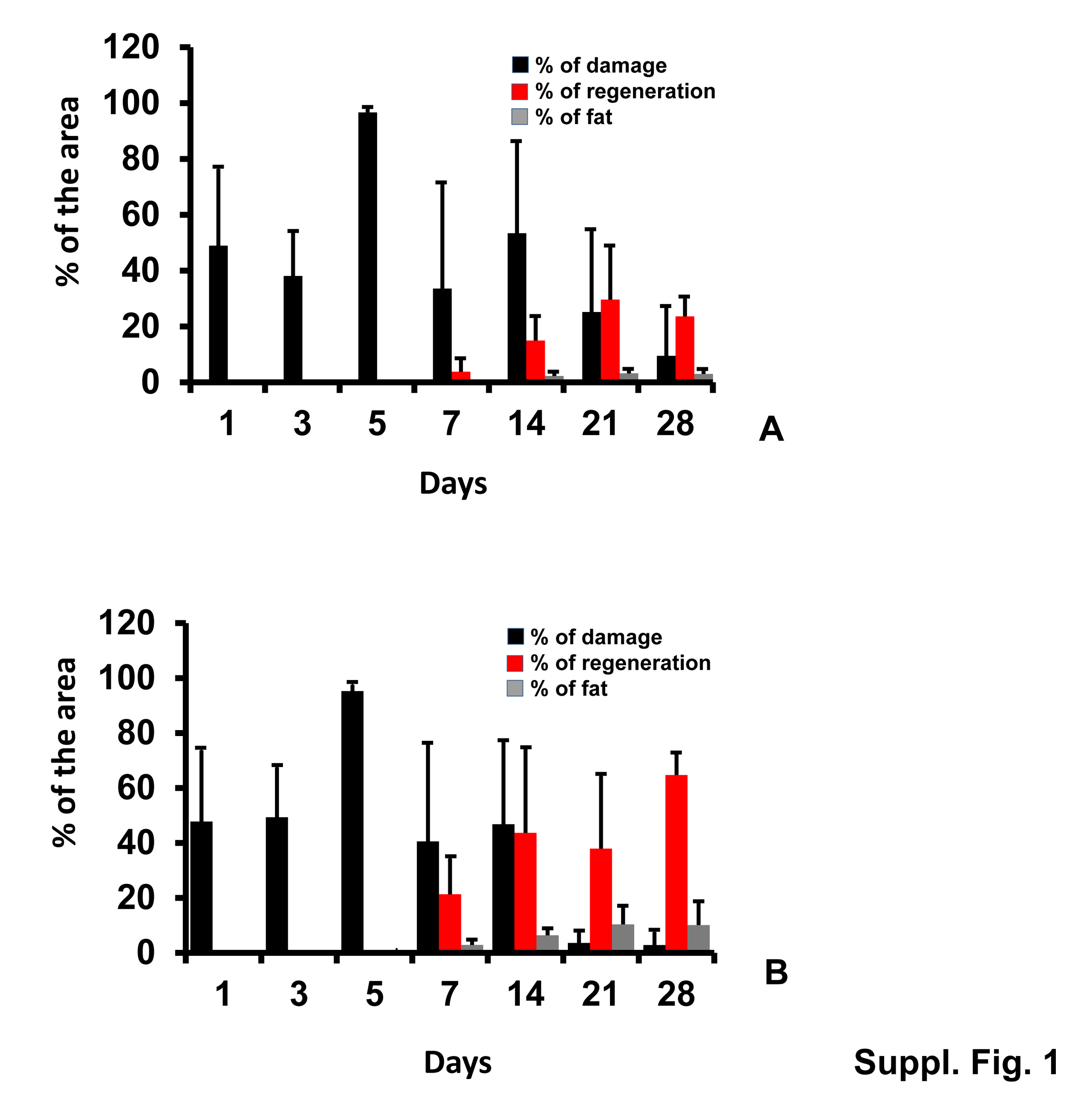

Supplement: S1 Fig — Gastrocnemius and Tibialis anterior muscles were harvested at the indicated times after Femoral artery dissection. Morphometric analysis was carried out on Hematoxilin/Eosin stained sections. The bar graphs show the percentage areas of necrotic, adipose or regenerating tissue. A) Gastrocnemius muscles; B) Tibialis anterior muscles. (TIF) [file pone.0142111.s001.tif]

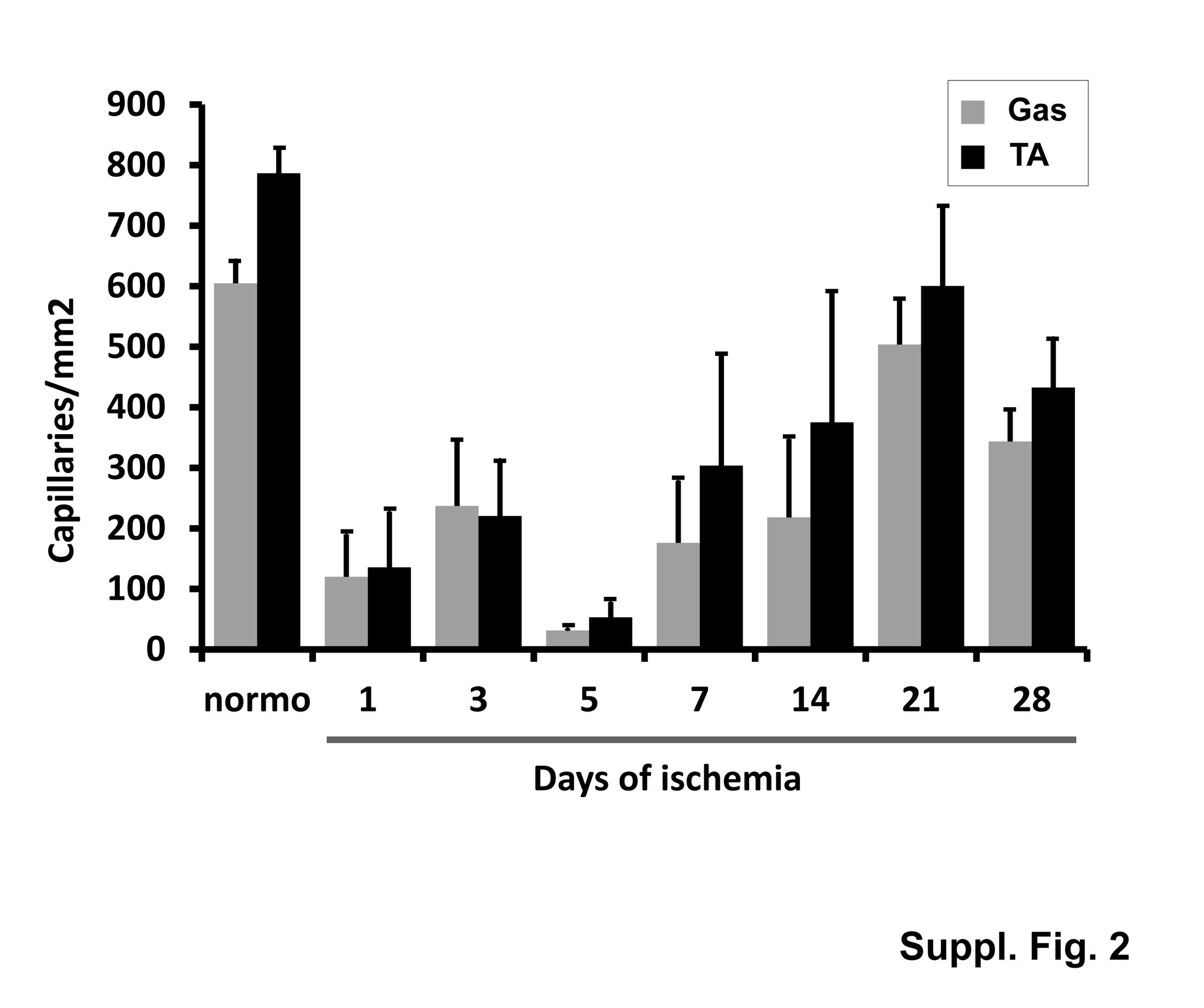

Supplement: S2 Fig — The bar graph represents the quantification of capillaries/mm2 of both Gastrocnemius (Gas) and Tibialis anterior (TA) muscles. (TIF) [file pone.0142111.s002.tif]

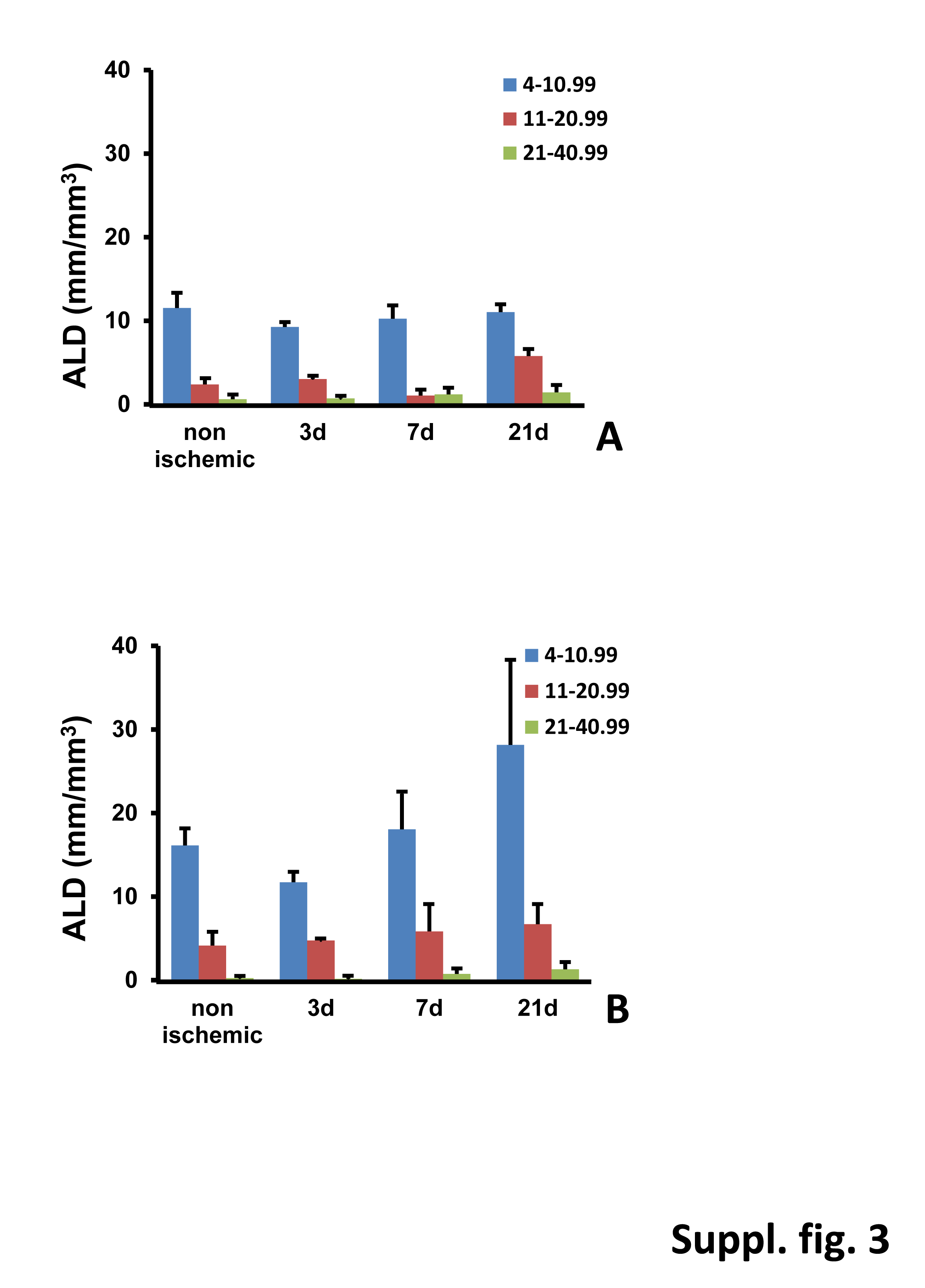

Supplement: S3 Fig — The bar ghraphs represent the ALD of Gastrocnemius (A) and Tibialis anterior (B) muscles. Arterioles were identified by α—smooth muscle actin (α—SMA) staining and were classified on the basis of the minimum external diameter in three different ranges: 4–10.99 μm, 11–20.99 μm and 21–40.99 μm. No statistically significant differences were observed (n = 3). (TIF) [file pone.0142111.s003.tif]

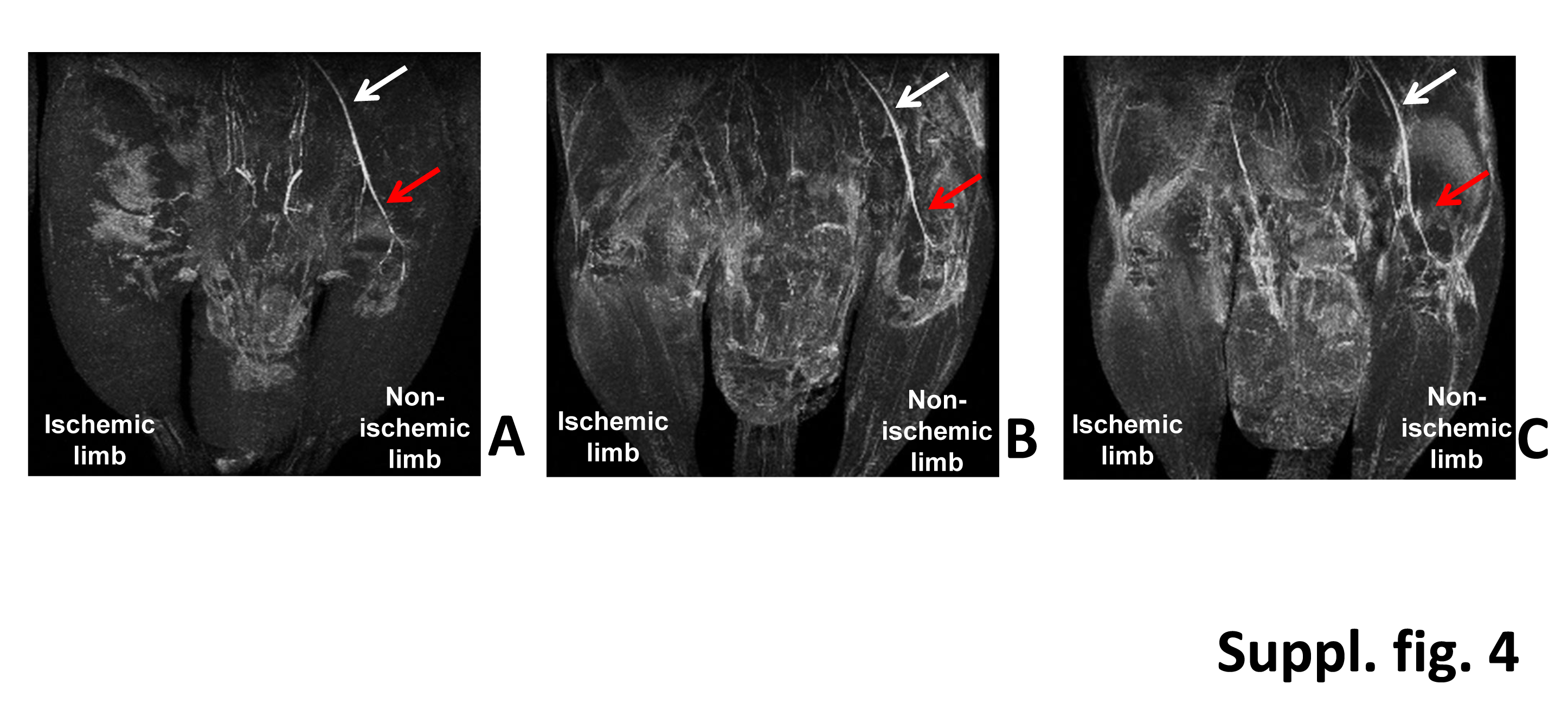

Supplement: S4 Fig — The pictures show coronal 3D FLASH images of both ischemic and non-ischemic limb of low-responding mice performed at 1 day after surgery. External Iliac (white arrows) and femoral artery (red arrows) are readily detectable in the right non-ischemic limb and completely undecteable in the left ischemic limb, demonstrating that surgery was carried out successfully in each ischemia resistant mice. A) Mouse m513; B) Mouse m588; C) Mouse m593. (TIF) [file pone.0142111.s004.tif]
