# Supplementary material for: Magnetic Resonance Imaging Allows the Evaluation of Tissue Damage and Regeneration in a Mouse Model of Critical Limb Ischemia
Source: PLoS One. 2015 Nov 10;10(11):e0142111. doi: 10.1371/journal.pone.0142111 (PMC4640853; doi:10.1371/journal.pone.0142111)
Supplement: S1 Table — MRI parameters of Tibialis anterior (TA) and Gastrocnemius (Gas) muscles, both ischemic (I) and non-ischemic (NI) measured after femoral artery dissection. Results are reported as average values of T2-rt (msec), FA (arbitrary units) and K-trans (min-1). The statistical significance (P) was calculated by Student’s T test comparing ischemic and non-ischemic controlateral muscles. (PDF) [file pone.0142111.s005.pdf]

		Day 1	Day 3	Day 5	Day 7	Day 14	Day 21	Day28
**T2-rt (msec)**	**Gas I**	39.49	39.13	38.58	36.84	35.62	33.84	33.13
	**Gas NI**	29.16	29.37	30.03	29.40	29.59	29.39	29.07
	P	0.002	0.004	0.009	0.02	0.07	0.12	0.14
	**TA I**	44.09	45.92	47.28	45.73	36.83	34.19	32.40
	**TA NI**	29.44	30.03	29.75	29.65	28.80	28.61	28.20
	P	0.00003	0.0001	0.0002	0.00006	0.003	0.03	0.12
**F.A. (a.u.)**	**Gas I**	0.18	0.18	0.22	0.22	0.24	0.25	0.25
	**Gas NI**	0.25	0.24	0.25	0.25	0.24	0.24	0.24
	P	0.006	0.01	0.13	0.32	0.73	0.64	0.72
	**TA I**	0.14	0.15	0.21	0.25	0.28	0.31	0.30
	**TA NI**	0.26	0.26	0.27	0.27	0.28	0.26	0.27
	P	0.000006	0.0004	0.03	0.60	0.92	0.02	0.07
**K-trans (min^{-1})**	**Gas I**	0.022	0.036	0.034	0.043	0.039	0.057	0.041
	P	0.00001	0.4	0.7	0.8	0.6	0.4	0.3
	**TA I**	0.030	0.037	0.020	0.060	0.059	0.071	0.074
	P	0.001	0.02	0.1	0.4	0.7	0.04	0.3

S 1 Table MRI parameters of Tibialis anterior (TA) and Gastrocnemius (Gas) muscles, both ischemic (I) and non-ischemic (NI) measured after femoral artery dissection. Results are reported as average values of T2-rt (msec), FA (arbitrary units) and K-trans (min^{-1}). The statistical significance (P) was calculated by Student's T test comparing ischemic and non-ischemic controlateral muscles.
